# l-DOPA and Freezing of Gait in Parkinson’s Disease: Objective Assessment through a Wearable Wireless System

**DOI:** 10.3389/fneur.2017.00406

**Published:** 2017-08-14

**Authors:** Antonio Suppa, Ardian Kita, Giorgio Leodori, Alessandro Zampogna, Ettore Nicolini, Paolo Lorenzi, Rosario Rao, Fernanda Irrera

**Affiliations:** ^1^Department of Neurology and Psychiatry, Sapienza University of Rome, Rome, Italy; ^2^IRCCS Neuromed Institute, Pozzilli, Italy; ^3^Department of Information Engineering, Electronics and Telecommunication, Sapienza University of Rome, Rome, Italy

**Keywords:** Parkinson’s disease, freezing of gait, wireless sensors, l-DOPA, inertial measurement unit, gait analysis

## Abstract

Freezing of gait (FOG) is a leading cause of falls and fractures in Parkinson’s disease (PD). The episodic and rather unpredictable occurrence of FOG, coupled with the variable response to l-DOPA of this gait disorder, makes the objective evaluation of FOG severity a major clinical challenge in the therapeutic management of patients with PD. The aim of this study was to examine and compare gait, clinically and objectively, in patients with PD, with and without FOG, by means of a new wearable system. We also assessed the effect of l-DOPA on FOG severity and specific spatiotemporal gait parameters in patients with and without FOG. To this purpose, we recruited 28 patients with FOG, 16 patients without FOG, and 16 healthy subjects. In all participants, gait was evaluated clinically by video recordings and objectively by means of the wearable wireless system, during a modified 3-m Timed Up and Go (TUG) test. All patients performed the modified TUG test under and not under dopaminergic therapy (ON and OFF therapy). By comparing instrumental data with the clinical identification of FOG based on offline video-recordings, we also assessed the performance of the wearable system to detect FOG automatically in terms of sensitivity, specificity, positive and negative predictive values, and finally accuracy. TUG duration was longer in patients than in controls, and the amount of gait abnormalities was prominent in patients with FOG compared with those without FOG. l-DOPA improved gait significantly in patients with PD and particularly in patients with FOG mainly by reducing FOG duration and increasing specific spatiotemporal gait parameters. Finally, the overall wireless system performance in automatic FOG detection was characterized by excellent sensitivity (93.41%), specificity (98.51%), positive predictive value (89.55%), negative predictive value (97.31%), and finally accuracy (98.51%). Our study overall provides new information on the beneficial effect of l-DOPA on FOG severity and specific spatiotemporal gait parameters as objectively measured by a wearable sensory system. The algorithm here reported potentially opens to objective long-time sensing of FOG episodes in patients with PD.

## Introduction

Freezing of gait (FOG) is an episodic gait disorder with the paroxysmal interruption of stride or marked reduction in forward feet progression (1), severely affecting quality of life and increasing risk of falls and fractures in patients with Parkinson’s disease (PD) (2, 3). In patients with PD, the pathophysiological investigation of FOG is rather challenging since FOG is crucially influenced by a number of cognitive, attentional, emotional, and even ecological factors (4–6). The current clinical evaluation of FOG severity is mainly based on patients’ subjective self-reported data that are largely affected by recall bias, thus precluding a clear interpretation of this disorder (7). A further important aspect in the clinical management of PD patients with FOG concerns the response of FOG to l-DOPA that is known to be rather complex and unpredictable. Although FOG most commonly manifests in patients not under dopaminergic treatment, in a number of patients with PD, FOG may persist or even worsen after acute l-DOPA administration (8–12). The objective evaluation of FOG and the response to l-DOPA are therefore critical clinical challenges with relevant impact in the current therapeutic management of patients with PD.

Over recent years, wearable technologies based on inertial measurement units (IMUs) have been increasingly used for the objective evaluation of specific motor symptoms including FOG in patients with PD (13, 14). Objective detection of FOG is currently achieved by using a various number of wearable IMUs with algorithms specifically designed, with time domain or frequency domain approaches, to detect or even predict FOG episodes in PD (13, 15–29). Previous studies have demonstrated that, due to the unobtrusive and wearable features, IMUs are optimal solutions for objective long-term monitoring of FOG in patients with PD, even in a domestic environment (13, 30). Hence, IMUs are also optimal candidates for objective evaluation of FOG response to l-DOPA in patients with PD.

So far, although a number of studies in PD have used IMUs to detect objectively FOG episodes, very few have objectively measured the effect of l-DOPA on FOG by using IMUs and only in relatively small cohorts of patients with PD (31). Moreover, none have used IMUs to compare spatiotemporal gait parameters between patients with and without FOG, under and not under dopaminergic therapy to clarify the pathophysiology of FOG (32–37). Filling in these gaps would help in better understanding the phenomenology of FOG in PD and its relationship with dopaminergic therapy. We here tested the hypothesis that l-DOPA influences spatiotemporal gait parameters differently in patients with and without FOG.

In this study, we investigated gait clinically and objectively, by means of IMUs, in a large cohort of patients with PD, with and without FOG, and compared all measures with those obtained in a cohort of healthy subjects. Participants were evaluated while performing a modified Timed Up and Go (TUG) test, a motor task designed to evaluate dynamic balance and functional mobility (19, 21, 24, 38). To clarify the effect of l-DOPA on FOG, we examined gait in patients with and without FOG, while performing the modified TUG test, under and not under dopaminergic therapy. We also specifically compared spatiotemporal gait parameters in patients with and without FOG, excluding FOG episodes from gait analysis. Finally, we examined the performance of a new algorithm for automatic FOG detection and thus suitable for objective long-term monitoring of FOG in patients with PD.

## Materials and Methods

### Subjects

Twenty-eight PD patients with FOG (18 men and 10 women, mean age 70.3 ± 7.30 years, mean disease duration 11.6 ± 6.70 years), 16 patients without FOG (14 men and 2 women, mean age 71.8 ± 6.45 years, mean disease duration 8.3 ± 5.37 years), and 16 age-matched healthy subjects (4 men and 12 women, mean age 69.7 ± 4.43 years) were recruited from the movement disorder outpatient clinic of the Department of Neurology and Psychiatry, Sapienza, University of Rome, and from IRCCS Neuromed Institute (Italy). The diagnosis of idiopathic PD was made according to current consensus criteria, and in all patients, the diagnosis was confirmed by follow-up clinical evaluations (39, 40). Patients with FOG were selected when showing a paroxysmal interruption of stride or marked reduction in forward feet progression during the clinical examination finalized to patients’ recruitment (1). Patients were clinically evaluated before starting each experimental session. The clinical assessment of motor symptoms included the following scales: Hoehn and Yahr (H&Y) (41) and Movement Disorders Society—Unified Parkinson’s Disease Rating Scale (MDS-UPDRS) part III (42). FOG and other axial symptoms were evaluated by using the FOG-Q (43) and the Postural Instability and Gait Difficulty (PIGD) score, calculated as the sum of items 2.12, 2.13, 3.10, 3.11, and 3.12 of the MDS-UPDRS (44). Cognitive evaluation included the Mini-Mental State Examination (MMSE) (45) and the Frontal Assessment Battery (FAB) (46). Mood and anxiety disorders were assessed by means of the Hamilton Rating Scale for Depression (HAM-D) (47) and the Beck Anxiety Inventory (BAI) (48). Inclusion criteria included a diagnosis of idiopathic PD, ability to walk independently, absence of comorbidities possibly affecting gait, including diabetes, rheumatic, or orthopedic disorders, and a MMSE score >24, thus excluding dementia. Patients were first studied after drug withdrawal for at least 12 h (OFF therapy) and then 1 h after the administration of their usual dopaminergic treatment (ON therapy) in the same experimental session. For each patient, the l-DOPA Equivalent Daily Doses (LEDDs) were calculated according to standardized procedures (49). None of the patients received other neuropsychiatric medications at the time of the study. Demographic and clinical features of patients, with and without FOG, are summarized in Table 1. All the subjects gave a written informed consent, and the experimental procedures have been approved by the institutional review board of Sapienza University of Rome, Italy, in agreement with the Declaration of Helsinki.

**Table 1:** Demographic and clinical features of PD patients with and without FOG.

						UPDRS-III									
Subjects	Sex	Age	Disease duration (years)	Phen	H&Y	ON	OFF	MMSE	FAB	HAM-D	BAI	Years after FOG onset	FOG-Q	PIGD	LEDDs
PD patients with FOG (*n* = 28)	18 M	70.3 ± 7.30	11.6 ± 6.70	24A/R, 4 Trem	2.6 ± 0.84	30.7 ± 13.70	39.7 ± 13.85	28.3 ± 19.60	14.6 ± 2.53	15.4 ± 7.76	12.7 ± 8.05	4.6 ± 4.51	15.4 ± 4.57	11.1 ± 4.38	797.7 ± 285.94
PD patients without FOG (*n* = 16)	14 M	71.8 ± 6.45	8.3 ± 5.37	11A/R, 5 Trem	2.0 ± 0.38	20.8 ± 10.54	28.2 ± 12.22	28.2 ± 1.90	15.1 ± 2.64	13.2 ± 5.69	11.7 ± 6.80			2.7 ± 2.54	700.4 ± 466.98

*Phen, phenotype; H&Y, Hoehn and Yahr scale; UPDRS-III, Unified Parkinson’s Disease Rating Scale—part III; MMSE, Mini-Mental State Evaluation; FAB, Frontal Assessment Battery; HAM-D, Hamilton Rating Scale for Depression; BAI, Beck Anxiety Inventory; FOG-Q, freezing of gait questionnaire; PIGD, Postural Instability and Gait Difficulty; LEDDs, l-Dopa Equivalent Daily Dose; PD, Parkinson’s disease*.

### Experimental Session

The experimental session consisted of anthropometric (height) and clinical data collection and then the execution of a motor task, a modified 3-m TUG test (38). Given the known unusual presentation of FOG, which is common in home environment but not under medical observation as in a research laboratory (50), in this study, the modified TUG test was carried out in a specific setting, reproducing a domestic environment. In detail, the route of 3 m of the modified TUG test involved the passage from a spacious room to a narrow corridor with the interposition of a door, reflecting a more ecological environment for FOG occurrence. Subjects were asked to rise from an armchair, walk forward at comfortable speed for 3 m, turn around, walk back to the chair, and sit down. Differently from the standard TUG, the point of turning was marked on the wall. Subjects were asked to perform the modified TUG test two times according to right-side or left-side turning (randomly selected) and both OFF and ON therapy (a total of two TUG tests for each healthy subject and two TUG tests for each subject with PD and state of therapy). To recognize specific FOG subtypes in relation to the effect of l-DOPA (i.e., OFF FOG, unresponsive FOG, pseudo-ON FOG, and finally ON FOG) (9), in patients manifesting poor response of FOG to their usual l-DOPA dose, we also used a supratherapeutic (double) dose of l-DOPA. The response of FOG to l-DOPA was clinically evaluated in terms of improvement in FOG duration during the modified TUG test. Patients were all videotaped while performing the modified TUG, with a camera standing at the end of the path in front of the patient. Video recordings of TUG trials allowed the offline evaluation by two independent neurologists, experts in movement disorders, blinded for state of therapy, serving as a gold standard for FOG detection. In this regard, FOG was defined as paroxysmal interruption of stride or marked reduction in forward feet progression. The two raters separately evaluated video recordings and assessed occurrence and duration of FOG episodes for each trial. FOG duration was defined as the sum of all FOG episodes (in seconds) within trials in the same state of therapy (OFF and ON). In case of discrepancy in FOG assessment between the two raters, a common assessment was performed to resolve the ambiguity. TUG duration was measured by a stopwatch considering the initial “GO” command and final patient’s contact with the chair at the end of modified TUG test.

### Wearable Sensing System

#### Hardware

**Figure 1:**
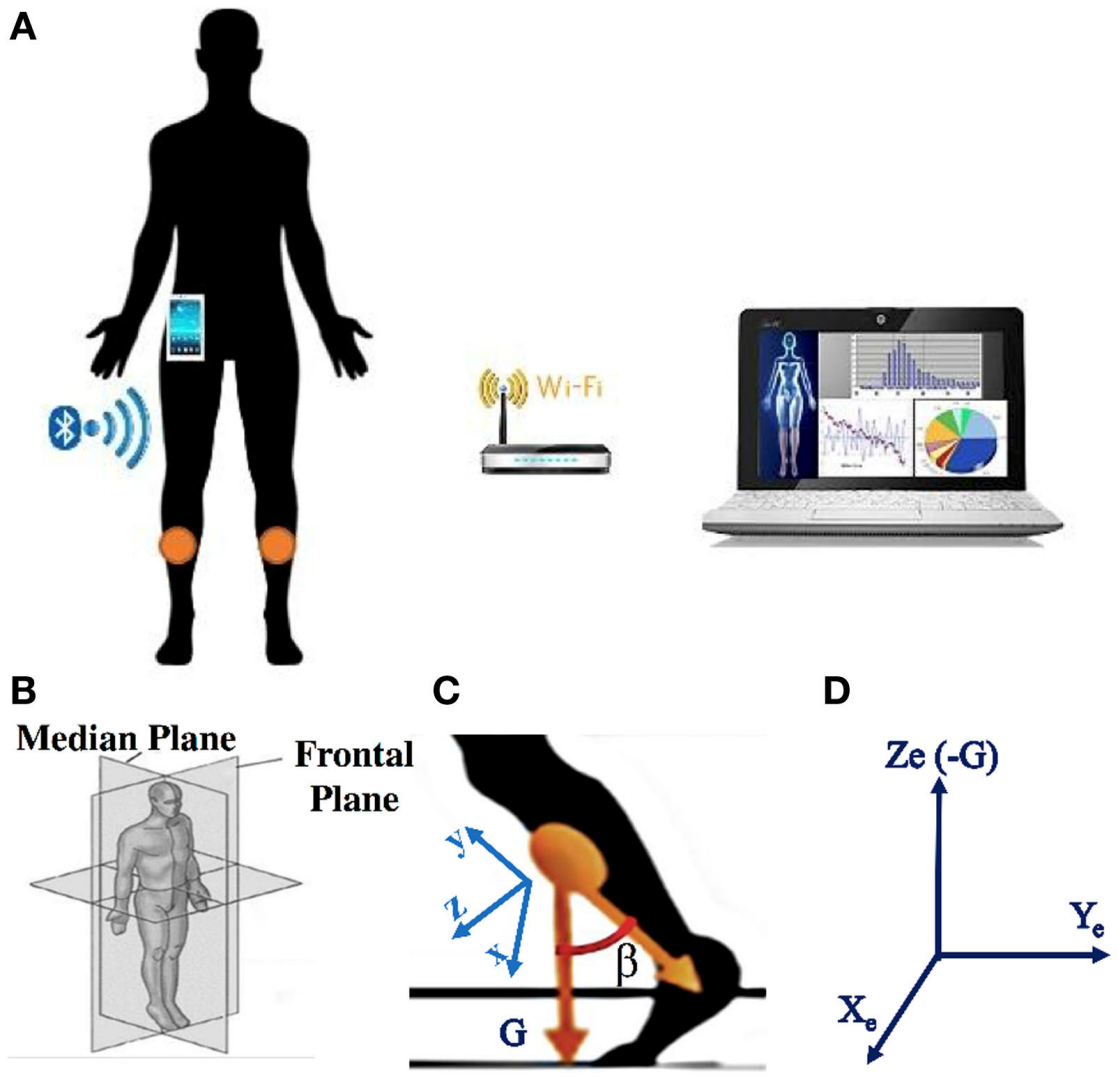
**(A)** Sketch of the system: the two sensors are positioned on the shins, a smartphone is used as portable receiver, a PC is connected to the smartphone *via* the Wi-fi or *via* Bluetooth; **(B)** representation of the reference systems where the gait takes place: median and frontal planes; **(C)** representation of the sensor reference system, with G the gravity direction; **(D)** representation of the earth reference system, in which the sensor reference system rotates.

The core of the wearable wireless system used for FOG detection and gait analysis consisted of two Inertial Measurements Units (IMUs) placed on the shins (Figure 1) (51, 52). The prototype IMU board was designed for processing signals in real time. It included the IMU LSM9DS0 integrating a ±16 g (g-force) 3D accelerometer and a ±2,000 dps 3D gyroscope in a 4 mm × 4 mm Land Grid Array package. Wireless communication was supported by a Bluetooth V3.0 module using the Serial Port Profile. The processing unit was an ultralow-power 32-bit microcontroller, with a 33.3 DMIPS peak computation capability and an extremely low power consumption scalable down to 233 uA/MHz. The Cortex™ M3 architecture along with the 32 MHz clock frequency makes this microcontroller suitable for advanced and low-power embedded computations. An USB 2.0 interface was present for battery recharge. The board including the battery has a total dimension of 25 mm × 30 mm × 4 mm. The offline postprocessing was performed by a PC, which realized an individual electronic diary where the information and data statistics in time are stored.

#### Algorithm for FOG Detection

*Ad hoc* algorithms were used to detect and classify FOG episodes in all participants. The recognition algorithm was based on a time domain analysis of sensor signals. The raw signals of accelerometer and gyroscope were fused together by using an orientation estimation algorithm proposed by Mahony et al. (53). To eliminate the gyroscope drift and to provide the sensor orientation in space, Mahony et al. (53) used a correction vector provided by a proportional integral (PI) controller, where the error vector ε driving the PI controller is determined from the previously estimated attitude and the accelerometer vector *a*. Mahony et al. (53) suggested to use ε = *a* × *d*, where *d* is the direction of the gravity vector as given by the estimated attitude. Regarding the PI controller, the value of the integral coefficient is *K_i_* = 0.0025, while the proportional coefficient is *K_p_* = 0.5. A quaternion-based representation of the limbs orientation and position was calculated, and a 3D vector representing the limbs was generated. The sampling frequency (*f_s_*) was 60 Hz (51) using a PC for the postprocessing, while it can be better set at 25 Hz (52) using a smartphone as a portable receiver. Reducing the sampling frequency has a benefit in that the number of transmitted data and operations per unit time becomes lower, thus improving the sensors and smartphone battery life. In turn, setting *f_s_* = 25 Hz does not present any drawbacks in the detection since the characteristic band of FOG in PD lies below 12 Hz (21). The sensors were positioned on the shins. Gait direction was in the median plane represented in Figure 1B. The x-y-z sensor reference system is sketched in Figure 1C. Figure 1D shows the Xe-Ye-Ze earth reference system in which the sensor reference system rotates. Ze coincides with negative G axis. The angle β sketched in Figure 1C is used for the FOG detection, and it is calculated as the angle formed between two 3D vectors: the negative y-axis and the gravity axis (G). It is worth noticing that the angle β is solid and, therefore, does not lie in the median plane. To detect FOG and calculate all the gait statistics, we need to analyze the projection of the β angle onto the median plane. In this way, any information on the rotation around the G axis is ignored. Eventual discontinuities of the β angle when it changes the sign, and consequent problems in angle derivation, can be easily overcome by conventional mathematical techniques.

The angular velocities ω_right_, ω_left_ obtained after the β angle derivation were used as the input for the FOG detection algorithm. That algorithm calculated the first-order low-pass filtered angular velocities. We defined as ω*_t_* and *k_t_*, respectively, as the right/left angular velocity and the low-pass filter measured at time *t, k_t-1_* the value of *k* at the previous step, α the smoothing coefficient set by the cutoff frequency (*f*_cutoff_):

(1a) $$K_{\text{right}}=\text{lowpass}(|\omega_{\text{right}}|)$$

(1b) $$K_{\text{left}}=\text{lowpass}(|\omega_{\text{left}}|)$$

(1c) $$K_{t}=(1-\alpha ).\omega_{t}+\alpha .k_{t-1}$$

(1d) $$\alpha =\;{\left(1+2\pi .f_{\text{cutoff}}/f_{s}\right)}^{-1},$$

where *f*_cutoff_ = 0.83 Hz (51). A *K* index was calculated and defined as:

(1e) $$K=K_{\text{right}}+K_{\text{left}}.$$

Once the values of T1 and T2 were fixed for a certain patient, they remained unchanged for the whole duration of the monitoring. To further implement algorithm and reduce false positives and negatives in FOG detection, especially during voluntary step shortening and slowing during turning, we also introduced a threshold *T*_turn_ and a *K*_turn_ index, defined as follows:

(2a) $$K_{\text{turn}}=\text{lowpass}(|\omega_{y}|)$$

(2b) $$K\prime =K+K_{\text{turn}}\text{ for }K_{\text{turn}}>T_{\text{turn}}$$

(2c) $$K\prime =K\text{ for }K_{\text{turn}}\leq T_{\text{turn}}.$$

A *K*_swing_ index was also introduced to definitely distinguish leg tremor due to body swinging and leg tremor during FOG, by using the following formula:

(3) $$K_{\text{swing}}=\text{lowpass}(|\omega_{Z}|).$$

**Figure 2:**
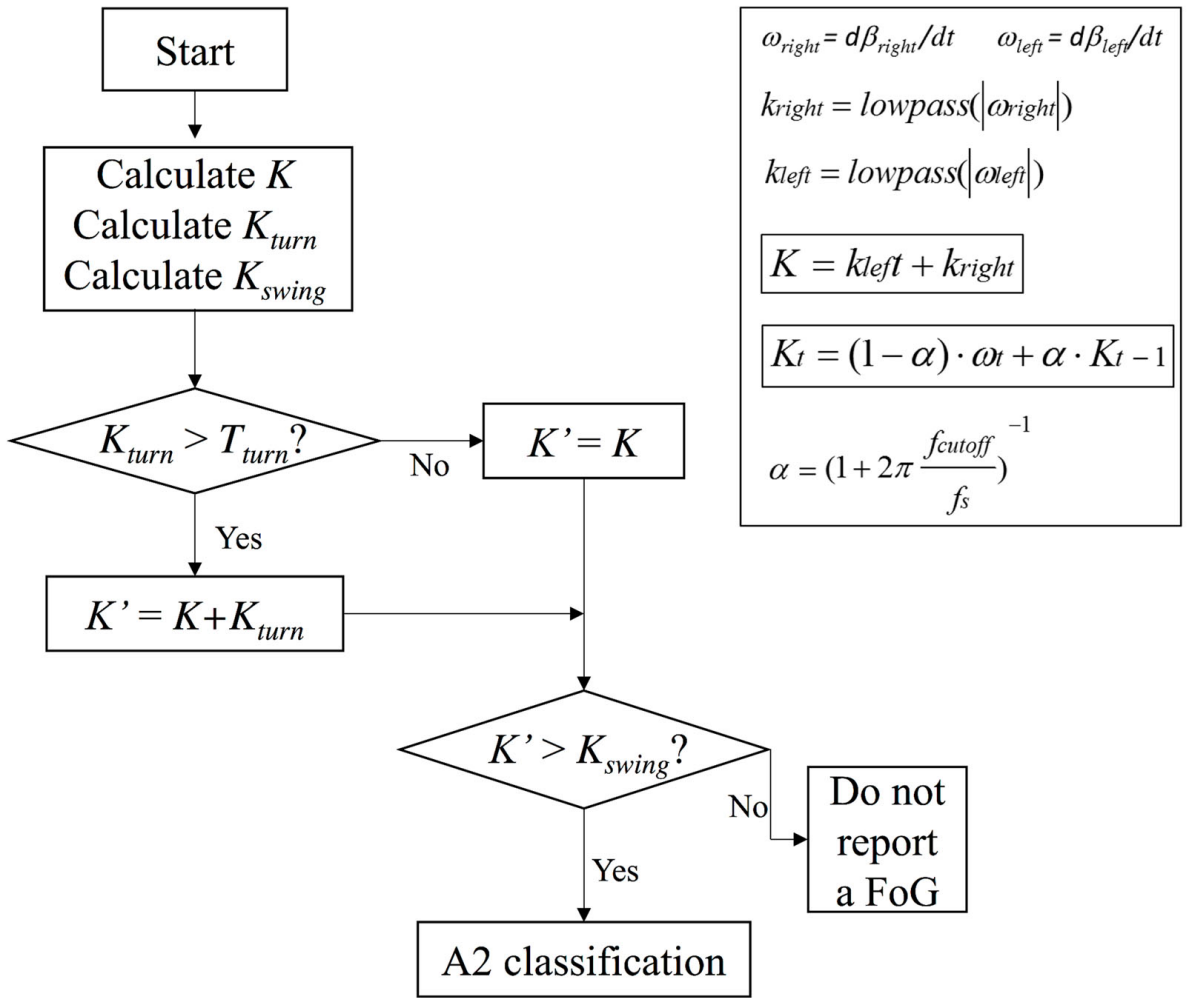
Block scheme of algorithm operations.

**Figure 3:**
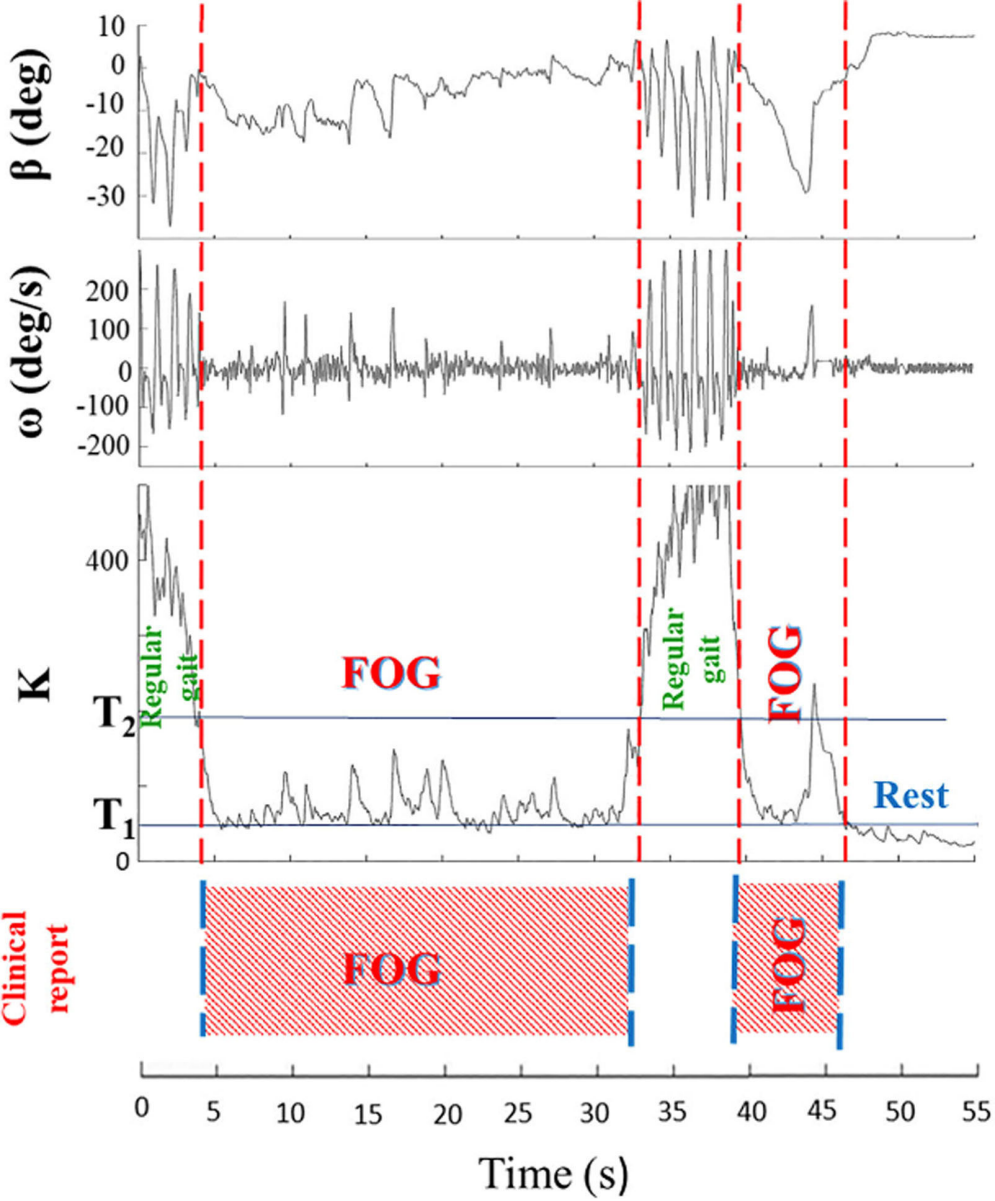
Angle β, angular velocity ω, *K* index, and clinical report during a sample test are shown. Clinical report allows to define two threshold values (T1 and T2) of *K* index, which automatically classify three stationary states: regular gait (*K* > T2), rest state (*K* < T1), and freezing of gait (FOG) episodes (T2 > *K* > T1). The wide dynamic range of the *K* index easily identifies distinct regions with different gait behaviors.

**Figure 4:**
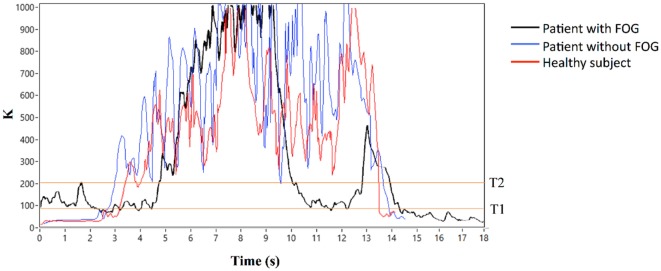
*K* index in a healthy subject, a Parkinson’s disease (PD) patient without freezing of gait (FOG), and a PD patient with FOG.

In summary, algorithm operations were the following: *K, K*_turn_, and *K*_swing_ indices were first calculated, *K*_turn_ index was compared with the threshold *T*_turn_ and, only in the case *K*_turn_ > *T*_turn_, *K*ʹ index was calculated (Eqs 2b and 2c). *K*ʹ index was then compared with *K*_swing_ index and, if *K*ʹ > *K*_swing_, the algorithm could exclude a body swing and classify a specific gait state. If *K*ʹ < *K*_swing_, leg movement was interpreted as a body swing (Figure 2). A practical application of the final algorithm is shown in Figure 3, where angle β, angular velocity ω, *K* index, and clinical report in a sample test are reported. During this sample test (Figure 3), very different behaviors of the angle β, angular velocity ω, and *K* index traces could be recognized. The traces of angle β and angular velocity ω clearly showed an oscillatory behavior during regular gait, such as in the time intervals 0–4 and 32–39 s, whereas they became flat traces during voluntary rest position when the subject was standing up at the end of the test. However, the traces of angle β and angular velocity ω were irregular and unpredictable when FOG episodes occurred, such as in the time intervals 4–32 and 39–46 s. On the contrary, the dynamic range of *K* index, that was rather wide, helped in the correct identification of every different gait behavior, also when FOG episodes occurred. In particular, clinical report of FOG episodes allowed to define two threshold values of *K* index (T1 and T2), which automatically classified three stationary states: regular gait, FOG state, and rest state, respectively, defined by *K* > T2, T2 > *K* > T1, and *K* < T1. Accordingly, in this specific sample test, clinical report agreed on the exact timing of three FOG episodes in the time intervals 4–32 and 39–46 s. An illustrative comparison of *K* index in a healthy subject, a PD patient without FOG, and a PD patient with FOG is shown in Figure 4.

#### Wireless Sensor-Based Gait Analysis

Gait analysis was performed in all participants except for seven patients with FOG. Gait analysis included the measurement of step velocity, stride length, stride time, and cadence as main spatiotemporal gait parameters in healthy subjects and in patients, OFF and ON therapy. Our analysis also included the evaluation of gait symmetry by comparing spatiotemporal gait parameters of the right and left leg. Step velocity (centimeters per second) was defined as the distance covered by the leg in time unit; stride length (centimeters), the distance between two consecutive heel strike of the same foot; stride time (seconds), the time from initial contact of one foot to subsequent contact of the same foot; and finally, cadence (steps per minute) was defined as the number of steps per minute. Spatiotemporal gait parameters were all expressed as the weighted average of right and left leg measures except for assessment of gait symmetry. In patients with FOG, sensor-based gait analysis included spatiotemporal gait parameters with the exclusion of FOG episodes. We have previously compared spatiotemporal gait parameters measured by means of our IMUs and by a standardized gait analysis lab based on an optoelectronic system (SMART analyzer motion system; BTS Bioengineering, Milan, Italy), and we have calculated a maximum error of ±2% (data not shown).

### Statistical Analysis

The Mann–Whitney *U* test was used to compare anthropometric data (height) in all participants and clinical features (age, disease duration, H&Y, UPDRS-III OFF and ON therapy, MMSE, FAB, HAM-D, BAI, PIGD score, and dopaminergic treatment as calculated by LEDDs), between patients with and without FOG. The Mann–Whitney *U* test was also used to compare TUG duration between healthy subjects and the whole group of patients. Finally, the Mann–Whitney *U* test was used to compare TUG duration in healthy subjects and in patients with and without FOG, OFF and ON therapy. The Wilcoxon signed-rank test was used to investigate the effect of dopaminergic treatment on UPDRS-III scores and TUG duration in patients with and without FOG and finally on FOG duration in patients with FOG. The Wilcoxon signed-rank test was also used to compare the number of FOG episodes at gait initiation, during straight passage through a narrow space, during turning, and finally during turn-to-sit, in patients with PD, OFF and ON therapy. Unpaired Student’s *t*-test was used to compare spatiotemporal gait parameters (step velocity, stride length, stride time, and cadence) between healthy subjects and patients with and without FOG, OFF and ON therapy. To compare all spatiotemporal gait parameters between patients with and without FOG, OFF and ON therapy, we used separate between-group analyses of variances (ANOVAs) with factors “Group” (patients with versus patients without FOG) and “dopaminergic therapy” (patients OFF versus ON therapy) as main factors of analysis. To evaluate gait symmetry in patients with and without FOG, OFF and ON therapy, we also used separate between-group ANOVAs with factors “Group” and “Side” (right versus left leg) as main factors of analysis. Tukey Honestly Significant Difference test was used for all *post hoc* analyses. Finally, Spearman rank correlation test was used to assess correlation between patients’ clinical features, FOG severity (as measured by years after FOG onset, scores at FOG-Q, FOG duration during TUG), TUG duration, and spatiotemporal gait parameters, in patients with and without FOG, OFF and ON therapy.

*P* values less than 0.05 were considered to indicate statistical significance.

The performance of the wearable sensing system to identify FOG episodes (presence or absence) was evaluated in terms of sensitivity (SE), specificity (SP), positive predictive value (PPV), negative predictive value (NPV), and accuracy (ACC) compared to the clinical identification of FOG based on offline video-recordings (gold standard).

## Results

The Mann–Whitney *U* test showed comparable anthropometric data (height) in healthy subjects and in patients with and without FOG (*P* > 0.05 for all comparisons) and comparable age, disease duration, and MMSE, FAB, HAM-D, and BAI scores (*P* > 0.05 for all comparisons) in patients with and without FOG. Conversely, the Mann–Whitney *U* test showed higher H&Y (*z* = −2.06; *P* = 0.045), UPDRS-III OFF (*z* = −2.42; *P* = 0.02) and UPDRS-III ON (*z* = −2.45; *P* = 0.001), PIGD scores (*z* = 5.01; *P* < 0.001), and finally, LEDDs (*z* = −2.1; *P* = 0.04) in patients with FOG than in those without FOG. The Wilcoxon signed-rank test showed that dopaminergic treatment improved UPDRS-III scores in the whole patients group (*z* = 5.45; *P* < 0.001) as well as in patients with FOG (*z* = 4.22; *P* < 0.001) and without FOG (*z* = 3.52; *P* < 0.001).

### Modified TUG Test

Clinical assessment of video recordings reported that 25 of 28 patients with definite FOG manifested at least 1 FOG episode while performing the modified TUG test, overall experiencing 152 FOG episodes (102 FOG episodes OFF therapy and 50 ON therapy). The Wilcoxon signed-rank test showed comparable number of FOG episodes in patients OFF and ON therapy at gait initiation, during straight passage through a narrow space, and finally during turn-to-sit (all *P* > 0.05 for all comparisons). By contrast, the number of FOG episodes differed during turning in patients OFF and ON therapy (*z* = −2.89; *P* < 0.01). During the modified TUG test in patients OFF therapy, FOG was elicited more frequently, respectively, by turning (41 FOG episodes, 40.2%), straight passage through a narrow space (21 FOG episodes, 20.6%), gait initiation (20 FOG episodes, 19.6%), and turn-to-sit (20 FOG episodes, 19.6%). Similarly, during the modified TUG test in patients ON therapy, FOG was elicited more frequently, respectively, by turning (22 FOG episodes, 44%), gait initiation (13 FOG episodes, 26%), turn-to-sit (10 FOG episodes, 20%), and straight passage through a narrow space (5 FOG episodes, 10%). Most of the patients with FOG manifested “OFF FOG” episodes because of the improvement or even the disappearance of FOG when ON therapy. Conversely, six patients presented “unresponsive FOG” given the absence of FOG improvement after administration of a supratherapeutic dose of l-DOPA. A single patient showed an ambiguous response to l-DOPA, with an apparent worsening of FOG, suggesting a case of “ON FOG.”

When comparing TUG duration in healthy subjects and in the whole group of patients with PD, the Mann–Whitney *U* test showed longer TUG duration in patients OFF (*z* = −3.34; *P* < 0.001) as well as ON therapy (*z* = −2.68; *P* < 0.01) than in controls. Patients with FOG had longer TUG duration compared with controls in OFF (*z* = −3.56; *P* < 0.001) and ON therapy (*z* = −3.23; *P* = 0.001). By contrast, patients without FOG had longer TUG duration compared with controls in OFF (*z* = −2.04; *P* < 0.04) but not in ON state of therapy (*z* = −1.06; *P* = 0.29). When comparing patients with and without FOG, TUG duration differed in OFF (*z* = −2.29; *P* = 0.02) but not in ON state of therapy (*z* = −1.76; *P* = 0.08). The Wilcoxon signed-rank test showed that dopaminergic treatment decreased TUG duration in the whole patients group (*z* = 5.34; *P* < 0.001) as well as in patients with (*z* = 4.28; *P* < 0.001) and without FOG (*z* = 3.52; *P* < 0.001). Finally, dopaminergic treatment also decreased FOG duration in patients with FOG (*z* = 2.27; *P* = 0.02) (Table 2).

**Table 2:** Average (±SD) Timed Up and Go (TUG) duration, total freezing of gait (FOG) duration, step velocity, stride length, stride time, and cadence in healthy subjects and Parkinson’s disease (PD) patients with and without FOG, OFF and ON therapy.

Subjects	State of therapy	TUG duration (s)	FOG duration (s)	Step velocity (cm/s)	Stride length (cm)	Stride time (s)	Cadence (steps/min)
Healthy subjects		18.6 ± 5.7		118.7 ± 37.17	77.7 ± 32.11	0.8 ± 0.10	111.2 ± 14.25
PD patients with FOG	OFF	49.9 ± 38.18	39.5 ± 63.50	76.0 ± 32.55	45.7 ± 28.49	0.8 ± 0.17	97.3 ± 18.18
	ON	31.4 ± 17.24	22.9 ± 48.37	96.6 ± 28.03	60.3 ± 20.74	0.8 ± 0.13	105.5 ± 22.67
PD patients without FOG	OFF	24.4 ± 7.79		71.4 ± 22.50	52.6 ± 21.25	0.9 ± 0.13	107.0 ± 18.83
	ON	21.5 ± 6.56		74.7 ± 19.61	48.0 ± 21.17	0.8 ± 0.17	106.2 ± 17.62

### Wireless Sensor-Based Gait Analysis

When comparing spatiotemporal gait parameters between healthy subjects and the whole group of PD patients, OFF and ON therapy, unpaired Student’s *t*-test showed higher step velocity and stride length in controls than in patients ON therapy (step velocity: *t* = 3.48; *P* = 0.001; stride length: *t* = 3.00; *P* = 0.004) and OFF therapy (step velocity: *t* = 4.76; *P* < 0.001; stride length: *t* = 3.49; *P* = 0.001), whereas stride time and cadence were comparable in the two study groups (*P* > 0.05 for all comparisons) (whole group of PD patients OFF: step velocity: 74.02 ± 26.62; stride length: 48.70 ± 25.76; stride time: 0.80 ± 0.16; cadence: 101.48 ± 19.10; PD patients ON: step velocity: 87.15 ± 26.99; stride length: 54.96 ± 21.82; stride time: 0.81 ± 0.15; cadence: 105.83 ± 20.58; Table 2; Figure S1 in Supplementary Material).

When comparing healthy subjects and patients with FOG, unpaired Student’s *t*-test again showed higher step velocity and stride length in controls than in patients with FOG in OFF (step velocity: *t* = 3.72; *P* < 0.001; stride length: *t* = 3.20; *P* = 0.003) and ON therapy (step velocity: *t* = 2.07; *P* < 0.05; stride length: *t* = 2.0; *P* < 0.05). Differently, stride time was comparable in controls and patients with FOG, OFF and ON therapy (*P* > 0.05 for all comparisons). Finally, patients with FOG had a lower cadence than controls in OFF (*t* = 2.52; *P* = 0.02), but not in ON therapy (*P* > 0.05) (Table 2).

When comparing healthy subjects and patients without FOG, unpaired Student’s *t*-test showed higher step velocity and stride length in controls than in patients in OFF (step velocity: *t* = 4.32; *P* < 0.001; stride length: *t* = 2.58; *P* = 0.01) and ON therapy (step velocity: *t* = 4.16; *P* < 0.005; stride length: *t* = 3.06; *P* = 0.005), whereas stride time and cadence were comparable in the two study groups (*P* > 0.05 for all comparisons) (Table 2).

When testing all spatiotemporal gait parameters in patients with and without FOG, OFF and ON therapy, between-group ANOVA showed a non-significant effect of the factor “Group” for step velocity (*F*_1.35_ = 2.48; *P* = 0.12), stride length (*F*_1.35_ = 0.14; *P* = 0.72), stride time (*F*_1.35_ = 1.66; *P* = 0.21), and cadence (*F*_1.35_ = 0.86; *P* = 0.36), whereas the factor “dopaminergic therapy” was significant only for step velocity (*F*_1.35_ = 15.6; *P* < 0.001), but not for stride length (*F*_1.35_ = 3.42; *P* = 0.07), stride time (*F*_1.35_ = 1.35; *P* = 0.94), and cadence (*F*_1.35_ = 1.21; *P* = 0.28). ANOVA also showed a significant interaction between factors “Group” and “dopaminergic therapy” for step velocity (*F*_1.35_ = 7.84; *P* < 0.01), stride length (*F*_1.35_ = 12.58; *P* < 0.001), and stride time (*F*_1.35_ = 4.78; *P* = 0.04), but not for cadence (*F*_1.35_ = 1.75; *P* = 0.19). *Post hoc* analysis demonstrated higher step velocity and stride length in patients with FOG than in patients without FOG ON (*P* < 0.01) but not OFF therapy (*P* > 0.05), whereas stride time was lower in patients with FOG than patients without FOG OFF (*P* = 0.003), but not ON therapy (*P* = 0.86). Dopaminergic therapy increased step velocity and stride length in patients with FOG (*P* = 0.001) but not in patients without FOG (*P* > 0.05), whereas it left stride time unchanged in patients with and without FOG (*P* = 0.39 and 0.46, respectively) (Table 2; Figure S2 in Supplementary Material).

When comparing gait symmetry in patients with and without FOG, OFF and ON therapy, ANOVA showed a non-significant effect of the factors “Group” and “Side” for step velocity, stride length, stride time, and cadence (*P* > 0.05 for all comparisons).

### Clinical-Behavioral Correlations

**Figure 5:**
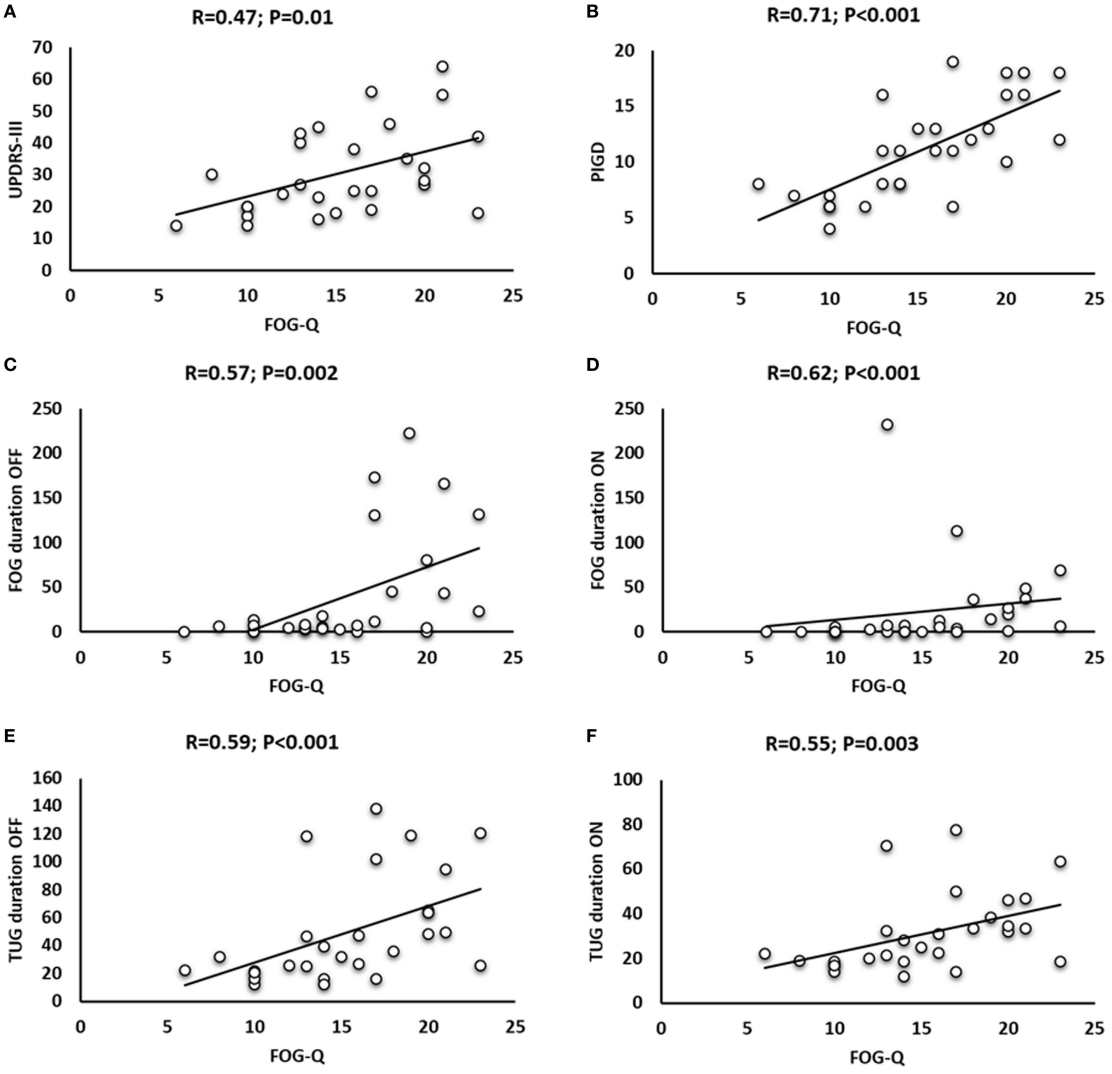
Correlation analysis in patients with freezing of gait (FOG) between freezing of gait questionnaire (FOG-Q) and Unified Parkinson’s Disease Rating Scale (UPDRS)-III ON therapy **(A)**, FOG-Q and Postural Instability and Gait Difficulty (PIGD) **(B)**, FOG-Q and FOG duration OFF **(C)** and ON therapy **(D)**, and FOG-Q and Timed Up and Go (TUG) duration OFF **(E)** and ON therapy **(F)**.

When assessing clinical-behavioral correlations in patients with FOG, Spearman rank correlation test found a positive correlation between years after FOG onset and LEDDs (*R* = 0.52; *P* = 0.005), and between FOG-Q and UPDRS-III ON therapy (*R* = 0.47; *P* = 0.01), PIGD scores (*R* = 0.71; *P* < 0.001), and FOG duration during TUG in patients OFF (*R* = 0.57; *P* = 0.002) and ON therapy (*R* = 0.62; *P* < 0.001). Spearman rank correlation test also found a positive correlation between FOG-Q and TUG duration in patients with FOG OFF (*R* = 0.59; *P* < 0.001) and ON therapy (*R* = 0.55; *P* = 0.003) and a positive correlation between TUG duration and FOG duration OFF (*R* = 0.59; *P* < 0.001) and ON therapy (*R* = 0.66; *P* < 0.001) (Figure 5).

### Performance of the Wearable System in FOG Detection

The performance of the wearable sensing system in automatic FOG detection, compared to the clinical identification of FOG based on offline video recordings (gold standard), showed the following average measures in 25 patients presenting FOG during the modified TUG test: SE 93.41%; SP 97.24%; PPV 89.55%; NPV 97.31%; and ACC 97.23% (Table 3), obtained with a latency of 400 ms. When considering PD patients not manifesting FOG during the modified TUG test and healthy subjects, the wearable sensing system showed 99.42% of SP and ACC. Accordingly, considering all the subjects participating to the study, the overall performance of the adopted wearable sensing system, using the proposed FOG detection algorithm, was the following: SE 93.41%; SP 98.51%; PPV 89.55%; NPV 97.31%; and ACC 98.51%.

**Table 3:** Performance of the wearable sensing system in FOG detection in PD patients presenting FOG during motor task.

Case	SE	SP	PPV	NPV	ACC
1	97.20	95.23	92.35	98.29	96.36
2	98.95	94.60	98.18	96.84	98.05
3	100.00	97.15	88.15	100.00	97.65
4	99.30	96.20	99.20	96.66	98.76
5	96.80	96.20	94.00	98.00	96.70
6	99.05	93.10	93.70	98.95	96.10
7	90.40	97.75	94.45	96.01	95.75
8	91.00	100.00	100.00	98.09	98.40
9	86.90	97.00	91.40	95.28	95.70
10	84.85	98.65	92.85	96.92	94.40
11	100.00	98.25	77.35	100.00	97.15
12	60.00	96.67	54.50	97.32	97.20
13	94.10	96.20	86.40	98.45	95.80
14	89.23	98.40	95.00	96.41	97.10
15	81.25	98.00	96.05	89.72	96.73
16	94.43	99.10	99.35	92.43	96.40
17	92.80	98.60	98.30	94.01	97.30
18	100.00	98.90	66.70	100.00	98.90
19	100.00	97.70	66.70	100.00	97.70
20	94.40	97.30	89.50	98.62	97.00
21	100.00	100.00	100.00	100.00	100.00
22	92.85	97.30	92.85	97.30	95.57
23	100.00	100.00	100.00	100.00	100.00
24	91.68	91.28	88.98	93.46	98.22
25	100.00	97.53	82.85	100.00	97.80
AV	93.41	97.24	89.55	97.31	97.23

*SE, sensitivity; SP, specificity; PPV, positive predictive value; NPV, negative predictive value; ACC, accuracy; AV, average; FOG, freezing of gait; FOG-Q, freezing of gait questionnaire; PD, Parkinson’s disease*.

## Discussion

We here report a detailed clinical and objective analysis of gait by means of IMUs, in a relatively large cohort of patients with PD, with and without FOG. We also provide new data on the effect of l-DOPA on clinical measures (modified TUG test) and spatiotemporal gait parameters, in patients with PD, with and without FOG. Finally, we here propose a new algorithm for automatic FOG detection in patients with PD and report the excellent performance of this new, unobtrusive, wearable sensing system.

Strict inclusion criteria allowed us to exclude a number of methodological factors possibly leading to misinterpretation of data. The clinical diagnosis of PD was made according to the current standardized criteria (39, 40), thereby reducing the possibility that our cohort included patients affected by neurological disorders other than PD such as atypical parkinsonism. We carefully excluded patients with comorbidities possibly affecting gait including diabetes, rheumatic, or orthopedic disorders. We enrolled patients without dementia as reflected by MMSE scores >24. To clarify motor response to dopaminergic treatments in patients with and without FOG, OFF and ON therapy, we examined each patient after 12 h of drug withdrawal (OFF), 1 h after acute administration of the best medical treatment (ON), and finally, in selected patients with poor response of FOG to l-DOPA, after a supratherapeutic (double) dose of l-DOPA (9).

The first finding in this study is that, although disease duration was comparable in the two patients’ subgroups (patients with and without FOG), patients with FOG had higher H&Y and UPDRS-III scores than patients without FOG, suggesting greater disease severity and progression. We also found higher LEEDs patients with FOG than in patients without FOG in agreement with previous observations (54–56). Finally, PIGD scores were also greater in patients with FOG than in patients without FOG and correlated significantly with FOG-Q scores confirming the association between the amount of axial impairment and severity of FOG in PD (55).

Previous clinical observations have raised the hypothesis that FOG reflects changes in frontal executive functions (57–60). However, when comparing patients enrolled in the present study, with and without FOG, we found similar FAB scores in the two patients’ subgroups, thus making unlikely the hypothesis that in our cohort of patients, a frontal disexecutive syndrome contributed significantly to the occurrence of FOG. Similarly, we found comparable HAM-D and BAI scores in patients with and without FOG, excluding that mood changes or anxiety disorders played a major role in the pathophysiology of FOG in our cohort of patients with FOG (61–64). Our clinical observations, however, do not exclude that possible changes in frontal executive functions and mood or anxiety disorders may further deteriorate FOG in patients with PD (57–64).

In line with previous studies (50, 65–68), during our modified TUG test, FOG occurred more frequently during postural transitions (turning and turn-to-sit), probably due to prominent axial impairment in patients with FOG, as demonstrated by higher H&Y and PIGD scores than patients without FOG. The other prevalent situation eliciting FOG occurrence during our modified TUG test was gait initiation, reflecting the complex motor and cognitive interaction to prepare and execute the first step, by performing adequate anticipatory postural adjustments (69, 70). Finally, the observation of a number of FOG episodes in the straight passage through a narrow space confirms the importance of ecological circumstances in triggering FOG (4, 5). This finding supports the hypothesis that, in patients with PD, abnormal visuospatial abilities lead to FOG occurrence by interfering with the online adjustment of gait pattern to environmental changes, such as the narrowing of the path (71–73). Finally, when examining the effect of dopaminergic treatment on the number of FOG episodes in patients with PD at gait initiation, during straight passage through a narrow space, during turning, and finally during turn-to-sit, we found that l-DOPA decreased significantly FOG episodes during turning likely by improving patients’ axial mobility. It is known that FOG is associated with akinetic-rigid phenotype (74), contributing to difficulties in change of direction.

### Gait in Patients with and without FOG, OFF Therapy

When clinically evaluating gait in the whole group of patients with PD and controls, while performing the modified TUG test, as expected, patients had longer TUG duration than controls confirming a number of previous observations (75, 76). When examining gait by means of our sensor-based analysis, we found that the longer TUG duration in patients with PD than in controls reflected changes in specific spatiotemporal gait parameters. Patients showed decreased step velocity and reduced stride length fully in agreement with several previous observations in PD (77–79). When comparing the whole group of patients with healthy subjects, our sensor-based gait analysis also showed similar cadence and stride time in patients and controls, confirming a previous hypothesis that in PD cadence increases to compensate for decreased step velocity and reduced stride length (80).

When we clinically evaluated gait, in patients with and without FOG, while performing the modified TUG test, TUG duration was significantly longer in patients with FOG than in those without FOG. However, when examining gait objectively, our sensor-based gait analysis disclosed comparable spatiotemporal gait parameters (when excluding FOG episodes) in the two patients’ subgroups. Our observation of comparable stride length in patients with and without FOG agrees with a previous study using IMUs (81) but apparently contrasts with others using pressure measurement systems, which showed shorter stride length in patients with FOG than in those without FOG (82, 83). The different methodology used to examine gait objectively, in patients with PD, including the path length and the measurement system likely explains such inconsistency. We therefore conclude that the longer TUG duration in patients with FOG than in those without FOG coupled with the observation of comparable spatiotemporal gait parameters (when excluding FOG episodes) in the two patients’ subgroups, specifically reflected the occurrence of FOG episodes. This conclusion is further supported by our observation of a positive correlation between TUG duration and FOG-Q scores as well as between TUG duration and FOG duration in patients with FOG. A further comment concerns the previously raised hypothesis that abnormal gait symmetry contributes to the pathophysiology of FOG (35, 84). When we compared spatiotemporal gait parameters in the right and left leg, in patients with and without FOG, our sensor-based gait analysis showed similar measures of gait symmetry in the two patients’ subgroups. We suggest that this inconsistency between our observations and those of Plotnik et al. (35, 84) might reflect the different degree of asymmetry in parkinsonian features (bradykinesia and rigidity) in the two cohorts of patients studied. In conclusion, our findings overall implying comparable spatiotemporal gait parameters, in patients with and without FOG, might agree with the hypothesis that FOG would reflect the paroxysmal disruption of gait rather than a progressive deterioration of motor control during gait (33–37). This hypothesis fits in well with the well-known existence of typical ecological circumstances implying emotional and attentional demanding tasks able to trigger FOG episodes abruptly (4, 5). Our findings also support the “cross-talk model” hypothesis (32, 85), which interprets FOG as a paroxysmal event. Accordingly, in PD, a functional interference in normally segregated cognitive, motor, and limbic circuits might generate a paroxysmal overactivity in basal ganglia output nuclei (Globus Pallus pars interna and Substantia Nigra pars reticulata) leading to abrupt deactivation of the pedunculopontine nucleus. Finally, a transient disruption of descending inputs from the pedunculopontine nucleus and other structures of the mesenchephalic locomotor region to spinal centers of gait would lead to FOG.

### Effect of l-DOPA on Gait in Patients with and without FOG

The clinical evaluation of gait in the whole group of patients with PD, while performing the modified TUG test under dopaminergic therapy, again showed longer TUG duration in patients than in healthy subjects. Hence, although TUG duration improved in patients under dopaminergic therapy, l-DOPA did not restore gait to normal levels in PD. This finding confirms previous observations reported in PD (86–88) and further support the hypothesis that l-DOPA improves the activation of neural circuits responsible for gait control in patients with PD (12, 89). When examining gait objectively, our sensor-based gait analysis again showed lower step velocity and stride length in patients than in controls, whereas stride time and cadence were still comparable in the two groups. This finding is fully in line with previous observations (89, 90), confirming that the inability to generate appropriate stride length is a crucial gait abnormality in PD, probably due to deficient internal cue production (12, 80).

The clinical evaluation of gait in patients with PD with and without FOG disclosed comparable TUG duration in the two patients’ subgroups. The observation that TUG duration was longer in patients with FOG than in those without FOG when OFF therapy, whereas it was similar in the two subgroups of subjects with PD when ON therapy, suggests that acute administration of l-DOPA improved gait prominently in patients FOG and such improvement mostly reflected reduced FOG occurrence. Our hypothesis confirms that in PD, FOG is mostly a dopaminergic-responsive gait disorder (3, 10, 91). In addition, when examining gait objectively, our sensor-based gait analysis showed that l-DOPA increased step velocity and stride length predominantly in patients with FOG compared to those without FOG, whereas stride time and cadence remained similar in the two patients’ subgroups. As previously discussed in patients OFF therapy, methodological factors likely explain the different stride length reported in patients with and without FOG, ON therapy, when comparing our study with those of Knobl et al. (82) and Barbe et al. (83). We speculate that, following acute administration of l-DOPA, our patients with FOG showed higher step velocity and stride length than patients without FOG probably as a result of increased attention on their walking pattern to support smooth gait and avoid FOG occurrence (80, 92). In conclusion, overall our findings suggest that the significant gait improvement observed in patients with FOG, ON therapy, reflects at least two factors, reduced FOG duration and improvement of specific spatiotemporal gait parameters.

A further comment concerns mechanisms possibly explaining why some of the patients with FOG here studied manifested a poor response to l-DOPA confirming a rather complex and unpredictable response of FOG to l-DOPA at least in a subgroup of patients with PD (9). Our analysis showed a positive correlation between years after FOG onset and LEDDs, suggesting that as PD progresses and FOG further deteriorates, the response of FOG to l-DOPA might progressively degrade, supporting the hypothesis that additional non-dopaminergic neurotransmitter systems contribute to the pathophysiology of FOG (93–97).

When considering the present findings, however, several limitations should be taken into account. In the present study, the male to female ratio slightly differed when comparing healthy subjects and subjects with PD, with and without FOG, possibly influencing specific spatiotemporal gait parameters (stride length and step velocity) here reported. Moreover, we did not evaluate frontal executive function by means of a detailed neuropsychological examination, thus possibly missing a subtle disexecutive syndrome in patients with FOG. The relatively limited path length of the modified TUG test here used to assess gait might have precluded us to evaluate subtle differences between patients with and without FOG in continuous gait abnormalities due to insufficient number of steps. Moreover, given that FOG often manifests during directional changes (4), our instrumental analysis focused on gait with the exclusion of FOG episodes might have overestimated the spatiotemporal gait parameters examined in patients with FOG. Finally, acceleration changes (gait initiation, turning, and turn-to-sit) due to our modified TUG test could also have influenced measures, thus requiring cautious consideration of absolute values of spatiotemporal gait parameters.

### Performance of the Algorithm

We here report systematic tests performed with a new algorithm designed for automatic detection of FOG episodes in patients with PD. Most of the previous studies reported algorithms for FOG detection working in the frequency domain, whereas our algorithm operates in the time domain. In frequency domain, some authors used the freezing index (FI) extrapolation implying the evaluation of the ratio between the power in the FOG band (2–6 Hz) associated to least leg tremor (51) and the power in the rest of the spectrum and comparing this ratio with defined thresholds. In this context, the first detection of FOG episodes was made by monitoring the body acceleration with a three-axis accelerometer (98). The authors applied fast Fourier transform, amplitude, and wavelet analysis performing an offline processing. Later, Moore et al. (21) analyzed offline the accelerometer data collected in 11 patients and detected the frequency components in the 3- to 8-Hz band during a FOG episode, which are not present during regular gait or at rest. Calculating the FI, their algorithm obtained 89% ACC and 89% SE in FOG detection. Following the algorithm proposed by Moore et al. (21), others developed a system for online FOG detection (17) containing three three-axial accelerometers and a wearable computer. The system was able to detect FOG episodes with user-dependent settings, exhibiting a SE of 88.6%, a SP of 92.4% evaluated on a sample of 10 patients, and a latency up to 2 s. Manual adjustment of the algorithm parameters was necessary to achieve optimal results. Other online FOG detection systems based on the FI extrapolation were presented in the studies by Jovanov et al. (16) and Djuric-Jovicic et al. (22). In the study by Jovanov et al. (16), the authors used a three-axis accelerometer and a wearable computer and detected FOG episodes with latency up to 580 ms. In the study by Djuric-Jovicic et al. (22), authors studied a sample of 12 PD patients and evaluated the SE in recognizing the occurrence of a FOG episode (reporting 100% of success), without evaluating the SE to timing and duration of each episode. Other methods of analysis in the frequency domain alternative to the FI extrapolation have been also developed including the algorithm proposed by Sijobert et al. (99) based on the evaluation of the step length and cadence. The authors made a comparison with the FI extrapolation and concluded that their algorithm appeared more accurate in recognizing FOG episodes. Conversely, in pure time domain, the signal amplitude is considered rather than the frequency band, so that a low-pass filter is needed to select the band of interest, and this factor is considered the main drawback of the time domain approach. The time domain analysis has the great advantage of performing a lower number of calculations, which turns into smaller power consumption and longer battery life. So far, very few studies with the pure time domain approach have been reported and among them, the work by Kwon et al. (25), which was based on the use of the root mean square of the accelerometer signal, and our previous work (52), which was based on the fusion of raw accelerometers and gyroscope signals. Both time domain approaches detected FOG episodes through a threshold method (25, 52). Kwon et al. (25) studied 20 patients with PD, obtaining a SE and a SP over 85%, whereas Kita et al. (52) studied 16 patients with PD, obtaining a SE and a SP over 94%. Finally, some work has been carried out in a combination of time and frequency domains, using different methods. Some authors used machine learning techniques (100, 101). SE and SP higher than 98% have been reported in Ref. (100) on a sample of 10 patients, with a latency up to 710 ms. In Pepa et al. (102), fuzzy logic algorithms were applied reporting good SE and SP in a group of 18 patients. More recently, Rezvanian and Lockhart (28) proposed using the continuous wavelet transform to define an index for identifying FOG episodes with good performances evaluated in a cohort of 10 patients. A final comment concerns that while the most used signal fusion algorithm for the calculation of sensor orientation in navigation systems is the Kalman filter (103), in our work, we opted for the algorithm proposed by Mahony et al. (53), which is less computationally expensive and therefore more convenient for wearable applications. By comparing the two algorithms, we got negligible difference in the orientation estimation with a noticeable benefit from the calculation load viewpoint. The reduction in the number of calculations allowed our system to detect FOG with a latency of only 400 ms, significantly lower than those reported in previous studies (16, 17, 100).

## Conclusion

We here report a detailed clinical and instrumental analysis of gait in a relatively large cohort of patients with PD, with and without FOG, showing that l-DOPA improves FOG duration and specific spatiotemporal gait parameters. We here also propose an unobtrusive wearable, wireless sensor system, including a new algorithm able to detect FOG episodes automatically, possibly helpful for long-term monitoring of FOG in patients with PD.

## Ethics Statement

All participants gave written informed consent and the experimental procedures have been approved by the institutional review board of Sapienza University of Rome, Italy, in agreement with the Declaration of Helsinki.

## Author Contributions

AS: conception, design, analysis, data interpretation, drafting, and critical revision of the manuscript. AK and AZ: design, analysis, data interpretation, and drafting of the manuscript. GL, PL, and RR: design, analysis, data interpretation, and critical revision of the manuscript. EN: analysis, data interpretation, and critical revision of the manuscript. FI: conception, design, data interpretation, and critical revision of the manuscript.

## Conflict of Interest Statement

The research was conducted in the absence of any commercial or financial relationships that could be construed as a potential conflict of interest.

## Abbreviations

ACC, accuracy; ANOVA, analysis of variance; BAI, Beck Anxiety Inventory; FAB, Frontal Assessment Battery; FI, freezing index; FOG, freezing of gait; FOG-Q, freezing of gait questionnaire; H&Y, Hoehn and Yahr; HAM-D, Hamilton Rating Scale for Depression; IMU, inertial measurement unit; MDS-UPDRS, Movement Disorders Society—Unified Parkinson’s Disease Rating Scale; MMSE, Mini-Mental State Examination; NPV, negative predictive value; PD, Parkinson’s disease; PPV, positive predictive value; SE, sensitivity; SP, specificity; PI, proportional integral; PIGD, postural instability and gait difficulty; TUG, Timed Up and Go test.

## References

[1] Nutt JG Bloem BR Giladi N Hallett M Horak FB Nieuwboer A. Freezing of gait: moving forward on a mysterious clinical phenomenon. Lancet Neurol (2011) 10:734–44. 10.1016/S1474-4422(11)70143-0 21777828 PMC7293393

[2] Bloem BR Hausdorff JM Visser JE Giladi N. Falls and freezing of gait in Parkinson’s disease: a review of two interconnected, episodic phenomena. Mov Disord (2004) 19:871–84. 10.1002/mds.20115 15300651

[3] Perez-Lloret S Negre-Pages L Damier P Delval A Derkinderen P Destée A Prevalence, determinants, and effect on quality of life of freezing of gait in Parkinson disease. JAMA Neurol (2014) 71:884–90. 10.1001/jamaneurol.2014.753 24839938

[4] Giladi N Freezing of gait. Clinical overview. Adv Neurol (2001) 87:191–7. 11347222

[5] Nieuwboer A Giladi N. The challenge of evaluating freezing of gait in patients with Parkinson’s disease. Br J Neurosurg (2008) 22:16–8. 10.1080/02688690802448376 19085348

[6] Barthel C Mallia E Deb B Bloem BR Ferraye MU The practicalities of assessing freezing of gait. J Parkinsons Dis (2016) 6:667–74. 10.3233/JPD-160927 27662331 PMC5088401

[7] Morris TR Cho C Dilda V Shine JM Naismith SL Lewis SJG A comparison of clinical and objective measures of freezing of gait in Parkinson’s disease. Parkinsonism Relat Disord (2012) 18:572–7. 10.1016/j.parkreldis.2012.03.001 22445248

[8] Schaafsma JD Balash Y Gurevich T Bartels AL Hausdorff JM Giladi N. Characterization of freezing of gait subtypes and the response of each to levodopa in Parkinson’s disease. Eur J Neurol (2003) 10:391–8. 10.1046/j.1468-1331.2003.00611.x 12823491

[9] Espay AJ Fasano A van Nuenen BF Payne MM Snijders AH Bloem BR “On” state freezing of gait in Parkinson disease: a paradoxical levodopa-induced complication. Neurology (2012) 78:454–7. 10.1212/WNL.0b013e3182477ec0 22262741 PMC3466608

[10] Fietzek UM Zwosta J Schroeteler FE Ziegler K Ceballos-Baumann AO. Levodopa changes the severity of freezing in Parkinson’s disease. Parkinsonism Relat Disord (2013) 19:894–6. 10.1016/j.parkreldis.2013.04.004 23642712

[11] Nonnekes J Snijders AH Nutt JG Deuschl G Giladi N Bloem BR. Freezing of gait: a practical approach to management. Lancet Neurol (2015) 14:768–78. 10.1016/S1474-4422(15)00041-1 26018593

[12] Smulders K Dale ML Carlson-Kuhta P Nutt JG Horak FB Pharmacological treatment in Parkinson’s disease: effects on gait. Parkinsonism Relat Disord (2016) 31:3–13. 10.1016/j.parkreldis.2016.07.006 27461783 PMC5048566

[13] Weiss A Herman T Giladi N Hausdorff JM. New evidence for gait abnormalities among Parkinson’s disease patients who suffer from freezing of gait: insights using a body-fixed sensor worn for 3 days. J Neural Transm (2015) 122:403–10. 10.1007/s00702-014-1279-y 25069586

[14] van Uem JM Maier KS Hucker S Scheck O Hobert MA Santos AT Twelve-week sensor assessment in Parkinson’s disease: impact on quality of life. Mov Disord (2016) 31:1337–8. 10.1002/mds.26676 27241524

[15] Moore ST MacDougall HG Ondo WG. Ambulatory monitoring of freezing of gait in Parkinson’s disease. J Neurosci Methods (2008) 167:340–8. 10.1016/j.jneumeth.2007.08.023 17928063

[16] Jovanov E Wang E Verhagen L Fredrickson M Fratangelo R Defog – a real time system for detection and unfreezing of gait of Parkinson’s patients. Conf Proc IEEE Eng Med Biol Soc (2009) 2009:5151–4. 10.1109/IEMBS.2009.5334257 19964859

[17] Bachlin M Plotnik M Roggen D Giladi N Hausdorff JM Tröster G A wearable system to assist walking of Parkinson’s disease patients. Methods Inf Med (2010) 49:88–95. 10.3414/ME09-02-0003 20011807

[18] Cole BT Roy SH Nawab SH. Detecting freezing-of-gait during unscripted and unconstrained activity. Conf Proc IEEE Eng Med Biol Soc (2011) 2011:5649–52. 10.1109/IEMBS.2011.6091367 22255621

[19] Morris TR Cho C Dilda V Shine JM Naismith SL Lewis SJ Clinical assessment of freezing of gait in Parkinson’s disease from computer-generated animation. Gait Posture (2013) 38:326–9. 10.1016/j.gaitpost.2012.12.011 23332192

[20] Takac B Català A Rodríguez Martín D van der Aa N Chen W Rauterberg M. Position and orientation tracking in a ubiquitous monitoring system for Parkinson disease patients with freezing of gait symptom. JMIR Mhealth Uhealth (2013) 1:e14. 10.2196/mhealth.2539 25098265 PMC4114461

[21] Moore ST Yungher DA Morris TR Dilda V MacDougall HG Shine JM Autonomous identification of freezing of gait in Parkinson’s disease from lower-body segmental accelerometry. J Neuroeng Rehabil (2013) 10:19. 10.1186/1743-0003-10-19 23405951 PMC3598888

[22] Djuric-Jovicic MD Jovicic NS Radovanovic SM Stankovic ID Popovic MB Kostić VS. Automatic identification and classification of freezing of gait episodes in Parkinson’s disease patients. IEEE Trans Neural Syst Rehabil Eng (2014) 22:685–94. 10.1109/TNSRE.2013.2287241 24235277

[23] Coste CA Sijobert B Pissard-Gibollet R Pasquier M Espiau B Geny C. Detection of freezing of gait in Parkinson disease: preliminary results. Sensors (Basel) (2014) 14:6819–27. 10.3390/s140406819 24740014 PMC4029660

[24] Yungher DA Morris TR Dilda V Shine JM Naismith SL Lewis SJ Temporal characteristics of high-frequency lower-limb oscillation during freezing of gait in Parkinson’s disease. Parkinsons Dis (2014) 2014:606427. 10.1155/2014/606427 25101189 PMC4101926

[25] Kwon Y Park SH Kim JW Ho Y Jeon HM Bang MJ A practical method for the detection of freezing of gait in patients with Parkinson’s disease. Clin Interv Aging (2014) 9:1709–19. 10.2147/CIA.S69773 25336936 PMC4199977

[26] Kim H Lee HJ Lee W Kwon S Kim SK Jeon HS Unconstrained detection of freezing of gait in Parkinson’s disease patients using smartphone. Conf Proc IEEE Eng Med Biol Soc (2015) 2015:3751–4. 10.1109/EMBC.2015.7319209 26737109

[27] Zach H Janssen AM Snijders AH Delval A Ferraye MU Auff E Identifying freezing of gait in Parkinson’s disease during freezing provoking tasks using waist-mounted accelerometry. Parkinsonism Relat Disord (2015) 21:1362–6. 10.1016/j.parkreldis.2015.09.051 26454703

[28] Rezvanian S Lockhart TE. Towards real-time detection of freezing of gait using wavelet transform on wireless accelerometer data. Sensors (Basel) (2016) 16:475. 10.3390/s16040475 27049389 PMC4850989

[29] Ahlrichs C Samà A Lawo M Cabestany J Rodríguez-Martín D Pérez-López C Detecting freezing of gait with a tri-axial accelerometer in Parkinson’s disease patients. Med Biol Eng Comput (2016) 54:223–33. 10.1007/s11517-015-1395-3 26429349

[30] Tao W Liu T Zheng R Feng H. Gait analysis using wearable sensors. Sensors (Basel) (2012) 12:2255–83. 10.3390/s120202255 22438763 PMC3304165

[31] Silva de Lima AL Evers LJ Hahn T Bataille L Hamilton JL Little MA Freezing of gait and fall detection in Parkinson’s disease using wearable sensors: a systematic review. J Neurol (2017) 264:1642–54. 10.1007/s00415-017-8424-0 28251357 PMC5533840

[32] Lewis SJG Barker RA. A pathophysiological model of freezing of gait in Parkinson’s disease. Parkinsonism Relat Disord (2009) 15:333–8. 10.1016/j.parkreldis.2008.08.006 18930430

[33] Nieuwboer A Dom R De Weerdt W Desloovere K Fieuws S Broens-Kaucsik E. Abnormalities of the spatiotemporal characteristics of gait at the onset of freezing in Parkinson’s disease. Mov Disord (2001) 16:1066–75. 10.1002/mds.1206 11748737

[34] Hausdorff JM Schaafsma JD Balash Y Bartels AL Gurevich T Giladi N. Impaired regulation of stride variability in Parkinson’s disease subjects with freezing of gait. Exp Brain Res (2003) 149:187–94. 10.1007/s00221-002-1354-8 12610686

[35] Plotnik M Giladi N Hausdorff JM. Bilateral coordination of walking and freezing of gait in Parkinson’s disease. Eur J Neurosci (2008) 27:1999–2006. 10.1111/j.1460-9568.2008.06167.x 18412621

[36] Chee R Murphy A Danoudis M Georgiou-Karistianis N Iansek R. Gait freezing in Parkinson’s disease and the stride length sequence effect interaction. Brain (2009) 132:2151–60. 10.1093/brain/awp053 19433440

[37] Plotnik M Giladi N Hausdorff JM. Is freezing of gait in Parkinson’s disease a result of multiple gait impairments? Implications for treatment. Parkinsons Dis (2012) 2012:459321. 10.1155/2012/459321 22288021 PMC3263650

[38] Podsiadlo D Richardson S The timed “up & go”: a test of basic functional mobility for frail elderly persons. J Am Geriatr Soc (1991) 39:142–8. 10.1111/j.1532-5415.1991.tb01616.x 1991946

[39] Gibb WR Lees AJ. The relevance of the Lewy body to the pathogenesis of idiopathic Parkinson’s disease. J Neurol Neurosurg Psychiatry (1988) 51:745–52. 10.1136/jnnp.51.6.745 2841426 PMC1033142

[40] Berardelli A Wenning GK Antonini A Berg D Bloem BR Bonifati V EFNS/MDS-ES/ENS (corrected) recommendations for the diagnosis of Parkinson’s disease. Eur J Neurol (2013) 20:16–34. 10.1111/ene.12022 23279440

[41] Hoehn M Yahr M Parkinsonism: onset, progression and mortality. Neurology (1967) 17:427–42. 10.1212/WNL.17.5.427 6067254

[42] Goetz C Tilley BC Shaftman SR Stebbins GT Fahn S Martinez-Martin P Movement disorder society-sponsored revision of the unified Parkinson’s disease rating scale (MDS-UPDRS): scale presentation and clinimetric testing results. Mov Disord (2008) 23:2129–70. 10.1002/mds.22340 19025984

[43] Giladi N Shabtai H Simon ES Biran S Tal J Korczyn AD. Construction of freezing of gait questionnaire for patients with Parkinsonism. Parkinsonism Relat Disord (2000) 6:165–70. 10.1016/S1353-8020(99)00062-0 10817956

[44] Stebbins GT Goetz CG Burn DJ Jankovic J Khoo TK Tilley BC. How to identify tremor dominant and postural instability/gait difficulty groups with the movement disorder society unified Parkinson’s disease rating scale: comparison with the unified Parkinson’s disease rating scale. Mov Disord (2013) 28:668–70. 10.1002/mds.25383 23408503

[45] Folstein MF Folstein SE McHugh PR Mini-mental state. A practical method for grading the cognitive state of patients for the clinician. J Psychiatr Res (1975) 12:189–98. 10.1016/0022-3956(75)90026-6 1202204

[46] Dubois B Slachevsky A Litvan I Pillon B. The FAB: a frontal assessment battery at bedside. Neurology (2000) 55:1621–6. 10.1212/WNL.55.11.1621 11113214

[47] Hamilton M A rating scale for depression. J Neurol Neurosurg Psychiatry (1960) 23:56–62. 10.1136/jnnp.23.1.56 14399272 PMC495331

[48] Beck AT Epstein N Brown G Steer RA An inventory for measuring clinical anxiety: psychometric properties. J Consult Clin Psychol (1988) 56:893–7. 10.1037/0022-006X.56.6.893 3204199

[49] Tomlinson CL Stowe R Patel S Rick C Gray R Clarke CE. Systematic review of levodopa dose equivalency reporting in Parkinson’s disease. Mov Disord (2010) 25:2649–53. 10.1002/mds.23429 21069833

[50] Snijders AH Haaxma CA Hagen YJ Munneke M Bloem BR. Freezer or non-freezer: clinical assessment of freezing of gait. Parkinsonism Relat Disord (2012) 18:149–54. 10.1016/j.parkreldis.2011.09.006 21968033

[51] Lorenzi P Rao R Romano G Kita A Irrera F Mobile devices for the real time detection of specific human motion disorders. IEEE Sens J (2016) 16:8220–7. 10.1109/JSEN.2016.2530944

[52] Kita A Lorenzi P Rao R Irrera F Reliable and robust detection of freezing of gait episodes with wearable electronic devices. IEEE Sens J (2017) 17:1–10. 10.1109/JSEN.2017.2659780

[53] Mahony R Hamel T Pflimlin JM Complementary filter design on the special orthogonal group SO (3). Proc. of the 44th IEEE CDC-ECC. Seville (2005). p. 1477–84.

[54] Giladi N Treves TA Simon ES Shabtai H Orlov Y Kandinov B Freezing of gait in patients with advanced Parkinson’s disease. J Neural Transm (2001) 108:53–61. 10.1007/s007020170096 11261746

[55] Giladi N McDermott MP Fahn S Przedborski S Jankovic J Stern M Freezing of gait in PD: prospective assessment in the DATATOP cohort. Neurology (2001) 56:1712–21. 10.1212/WNL.56.12.1712 11425939

[56] Macht M Kaussner Y Möller JC Stiasny-Kolster K Eggert KM Krüger HP Predictors of freezing in Parkinson’s disease: a survey of 6,620 patients. Mov Disord (2007) 22:953–6. 10.1002/mds.21458 17377927

[57] Giladi N Huber-Mahlin V Herman T Hausdorff JM. Freezing of gait in older adults with high level gait disorders: association with impaired executive function. J Neural Transm (2007) 114:1349–53. 10.1007/s00702-007-0772-y 17576512

[58] Amboni M Cozzolino A Longo K Picillo M Barone P. Freezing of gait and executive functions in patients with Parkinson’s disease. Mov Disord (2008) 23:395–400. 10.1002/mds.21850 18067193

[59] Vandenbossche J Deroost N Soetens E Spildooren J Vercruysse S Nieuwboer A Freezing of gait in Parkinson disease is associated with impaired conflict resolution. Neurorehabil Neural Repair (2011) 25:765–73. 10.1177/1545968311403493 21478498

[60] Cohen RG Klein KA Nomura M Fleming M Mancini M Giladi N Inhibition, executive function, and freezing of gait. J Parkinsons Dis (2014) 4:111–22. 10.3233/JPD-130221 24496099 PMC4028962

[61] Lieberman A. Are freezing of gait (FOG) and panic related? J Neurol Sci (2006) 248:219–22. 10.1016/j.jns.2006.05.023 16797596

[62] Giladi N Hausdorff JM. The role of mental function in the pathogenesis of freezing of gait in Parkinson’s disease. J Neurol Sci (2006) 248:173–7. 10.1016/j.jns.2006.05.015 16780886

[63] Martens KA Ellard CG Almeida QJ. Does anxiety cause freezing of gait in Parkinson’s disease? PLoS One (2014) 9:e106561. 10.1371/journal.pone.0106561 25250691 PMC4175083

[64] Martens KA Halla JM Gilata M Georgiadesa MJ Waltona CC Lewis SJG. Anxiety is associated with freezing of gait and attentional set-shiftingin Parkinson’s disease: a new perspective for early intervention. Gait Posture (2016) 49:431–6. 10.1016/j.gaitpost.2016.07.182 27513741

[65] Snijders AH Nijkrake MJ Bakker M Munneke M Wind C Bloem BR. Clinimetrics of freezing of gait. Mov Disord (2008) 23:468–74. 10.1002/mds.22144 18668628

[66] Spildooren J Vercruysse S Desloovere K Vandenberghe W Kerckhofs E Nieuwboer A. Freezing of gait in Parkinson’s disease: the impact of dual-tasking and turning. Mov Disord (2010) 25:2563–70. 10.1002/mds.23327 20632376

[67] Spildooren J Vercruysse S Heremans E Galna B Vandenbossche J Desloovere K Head-pelvis coupling is increased during turning in patients with Parkinson’s disease and freezing of gait. Mov Disord (2013) 28:619–25. 10.1002/mds.25285 23408374

[68] Mancini M Smulders K Cohen RG Horak FB Giladi N Nutt JG. The clinical significance of freezing while turning in Parkinson’s disease. Neuroscience (2017) 343:222–8. 10.1016/j.neuroscience.2016.11.045 27956066 PMC5289743

[69] Jacobs JV Nutt JG Carlson-Kuhta P Stephens M Horak FB. Knee trembling during freezing of gait represents multiple anticipatory postural adjustments. Exp Neurol (2009) 215:334–41. 10.1016/j.expneurol.2008.10.019 19061889 PMC3141813

[70] Delval A Tard C Defebvre L. Why we should study gait initiation in Parkinson’s disease. Neurophysiol Clin (2014) 44:69–76. 10.1016/j.neucli.2013.10.127 24502907

[71] Almeida QJ Lebold CA. Freezing of gait in Parkinson’s disease: a perceptual cause for a motor impairment? J Neurol Neurosurg Psychiatry (2010) 81:513–8. 10.1136/jnnp.2008.160580 19758982

[72] Nantel J McDonald JC Tan S Bronte-Stewart H. Deficits in visuospatial processing contribute to quantitative measures of freezing of gait in Parkinson’s disease. Neuroscience (2012) 221:151–6. 10.1016/j.neuroscience.2012.07.007 22796080

[73] Silveira CR Ehgoetz Martens KA Pieruccini-Faria F Bell-Boucher D Roy EA Almeida QJ. Disentangling perceptual judgment and online feedback deficits in Parkinson’s freezing of gait. J Neurol (2015) 262:1629–36. 10.1007/s00415-015-7759-7 25929667 PMC4503856

[74] Lamberti P Armenise S Castaldo V de Mari M Iliceto G Tronci P Freezing gait in Parkinson’s disease. Eur Neurol (1997) 38:297–301. 10.1159/000113398 9434089

[75] Kotagal V Albin RL Müller ML Koeppe RA Studenski S Frey KA Advanced age, cardiovascular risk burden, and timed up and go test performance in Parkinson disease. J Gerontol A Biol Sci Med Sci (2014) 69:1569–75. 10.1093/gerona/glu070 24864306 PMC4296117

[76] Son M Youm C Cheon S Kim J Lee M Kim Y Evaluation of the turning characteristics according to the severity of Parkinson disease during the timed up and go test. Aging Clin Exp Res (2017). 10.1007/s40520-016-0719-y 28220396

[77] Morris ME Iansek R Matyas TA Summers JJ. Ability to modulate walking cadence remains intact in Parkinson’s disease. J Neurol Neurosurg Psychiatry (1994) 57:1532–4. 10.1136/jnnp.57.12.1532 7798986 PMC1073238

[78] Morris ME Iansek R Matyas TA Summers JJ. The pathogenesis of gait hypokinesia in Parkinson’s disease. Brain (1994) 117:1169–81. 10.1093/brain/117.5.1169 7953597

[79] Nieuwboer A De Weerdt W Dom R Peeraer L Lesaffre E Hilde F Plantar force distribution in parkinsonian gait: a comparison between patients and age-matched control subjects. Scand J Rehabil Med (1999) 31:185–92. 10.1080/003655099444533 10458317

[80] Morris ME Iansek R Matyas TA Summers JJ. Stride length regulation in Parkinson’s disease. Normalization strategies and underlying mechanisms. Brain (1996) 119:551–68. 10.1093/brain/119.2.551 8800948

[81] Peterson DS Fling BW Mancini M Cohen RG Nutt JG Horak FB Dual task interference and brain structural connectivity in people with Parkinson’s disease who freeze. J Neurol Neurosurg Psychiatry (2015) 86:786–92. 10.1136/jnnp-2014-308840 25224677 PMC4363035

[82] Knobl P Kielstra L Almeida Q. The relationship between motor planning and freezing of gait in Parkinson’s disease. J Neurol Neurosurg Psychiatry (2012) 83:98–101. 10.1136/jnnp-2011-300869 21836031

[83] Barbe MT Amarell M Snijders AH Florin E Quatuor EL Schönau E Gait and upper limb variability in Parkinson’s disease patients with and without freezing of gait. J Neurol (2014) 261:330–42. 10.1007/s00415-013-7199-1 24305993

[84] Plotnik M Giladi N Balash Y Peretz C Hausdorff JM. Is freezing of gait in Parkinson’s disease related to asymmetric motor function? Ann Neurol (2005) 57:656–63. 10.1002/ana.20452 15852404

[85] Lewis SJ Shine JM. The next step: a common neural mechanism for freezing of gait. Neuroscientist (2016) 22:72–82. 10.1177/1073858414559101 25398230

[86] Morris S Morris ME Iansek R Reliability of measurements obtained with the timed “up & go” test in people with Parkinson disease. Phys Ther (2001) 81:810–8. 10.1093/ptj/81.2.810 11175678

[87] Rossi M Soto A Santos S Sesar A Labella T. A prospective study of alterations in balance among patients with Parkinson’s disease. Protocol of the postural evaluation. Eur Neurol (2009) 61:171–6. 10.1159/000189270 19129704

[88] Weiss A Herman T Plotnik M Brozgol M Maidan I Giladi N Can an accelerometer enhance the utility of the timed up & go test when evaluating patients with Parkinson’s disease? Med Eng Phys (2010) 32:119–25. 10.1016/j.medengphy.2009.10.015 19942472

[89] Curtze C Nutt JC Carlson-Kuhta P Mancini M Horak FB. Levodopa is a double-edged sword for balance and gait in people with Parkinson’s disease. Mov Disord (2015) 30:1361–70. 10.1002/mds.26269 26095928 PMC4755510

[90] Bayle N Patel AS Crisan D Guo LJ Hutin E Weisz DJ Contribution of step length to increase walking and turning speed as a marker of Parkinson’s disease progression. PLoS One (2016) 11:e0152469. 10.1371/journal.pone.0152469 27111531 PMC4844147

[91] Amboni M Stocchi F Abbruzzese G Morgante L Onofrj M Ruggieri S Prevalence and associated features of self-reported freezing of gait in Parkinson disease: the DEEP FOG study. Parkinsonism Relat Disord (2015) 21:644–9. 10.1016/j.parkreldis.2015.03.028 25899545

[92] Behrman AL Teitelbaum P Cauraugh JH. Verbal instructional sets to normalise the temporal and spatial gait variables in Parkinson’s disease. J Neurol Neurosurg Psychiatry (1998) 65:580–2. 10.1136/jnnp.65.4.580 9771792 PMC2170270

[93] Grimbergen YA Langston JW Roos RA Bloem BR. Postural instability in Parkinson’s disease: the adrenergic hypothesis and the locus coeruleus. Expert Rev Neurother (2009) 9:279–90. 10.1586/14737175.9.2.279 19210201

[94] Jankovic J. Atomoxetine for freezing of gait in Parkinson disease. J Neurol Sci (2009) 284:177–8. 10.1016/j.jns.2009.03.022 19361809

[95] Devos D Defebvre L Bordet R. Dopaminergic and non-dopaminergic pharmacological hypotheses for gait disorders in Parkinson’s disease. Fundam Clin Pharmacol (2010) 24:407–21. 10.1111/j.1472-8206.2009.00798.x 20163480

[96] Ferraye MU Debû B Fraix V Goetz L Ardouin C Yelnik J Effects of pedunculopontine nucleus area stimulation on gait disorders in Parkinson’s disease. Brain (2010) 133:205–14. 10.1093/brain/awp229 19773356

[97] Thevathasan W Cole MH Graepel CL Hyam JA Jenkinson N Brittain JS A spatiotemporal analysis of gait freezing and the impact of pedunculopontine nucleus stimulation. Brain (2012) 135:1446–54. 10.1093/brain/aws039 22396391 PMC3338924

[98] Han H Lee W Ahn TB Jeon BS Park KS Gait analysis for freezing detection in patients with movement disorder using three-dimensional acceleration system. Proc EMBS (2003) 2:1863–5. 10.1109/IEMBS.2003.1279781

[99] Sijobert B Denys J Coste CA Geny C IMU based detection of freezing of gait and festination in Parkinson’s disease. Proc of the IEEE 19th International IFESS 2014, Kuala Lumpur (2014). p. 1–3.

[100] Bonato P Sherrill D Standaert D Salles S Akay M Data mining techniques to detect motor fluctuations in Parkinson’s disease. 26th Annual Int. Conf. in Engineering in Medicine and Biology Society, IEEE, 2004. San Francisco, CA (2004). p. 4766–9. 10.1109/IEMBS.2004.1404319 17271375

[101] Mazilu S Hardegger M Zhu Z Roggen D Troster G Plotnik M Online detection of freezing of gait with smartphones and machine learning techniques. 6th Int. Conf. Pervasive Health, IEEE, 2012. San Diego, CA (2012). p. 123–30.

[102] Pepa L Ciabattoni L Verdini F Capecci M Ceravolo MG Smartphone based fuzzy logic freezing of gait detection in Parkinson’s disease. 10th Int. Conf. MESA, IEEE, 2014. Senigallia (2014). p. 1–6.

[103] Kalman RE A new approach to linear filtering and prediction problems. J Basic Eng (1960) 82:35–45. 10.1115/1.3662552

