# Supplementary material for: l-DOPA and Freezing of Gait in Parkinson’s Disease: Objective Assessment through a Wearable Wireless System
Source: Front Neurol. 2017 Aug 14;8:406. doi: 10.3389/fneur.2017.00406 (PMC5557738; doi:10.3389/fneur.2017.00406)
Supplement: Supplementary file 1 [file data_sheet_1.doc]

Supplementary Material

L-Dopa and Freezing of Gait in Parkinson’s Disease:

Objective Assessment Through a Wearable Wireless System

A. Suppa*, A. Kita, G. Leodori, A. Zampogna, E. Nicolini, P. Lorenzi, R. Rao and

F. Irrera

*** Correspondence:** Dr. Antonio Suppa, MD, PhD: [antonio.suppa@uniroma1.it](mailto:antonio.suppa@uniroma1.it)

**SUPPLEMENTARY FIGURES**

**Supplementary Figure 1:** Spatio-temporal gait parameters including Step Velocity (A), Stride Length (B), Stride Time (C) and finally Cadence (D) in healthy subjects (HS) and in the whole group of patients with Parkinson’s disease (PD), OFF and ON therapy.

**Supplementary Figure 2**: Spatio-temporal gait parameters including Step Velocity (A), Stride Length (B), Stride Time (C) and finally Cadence (D) in patients with Parkinson’s disease (PD), with (FOG+) and without FOG (FOG-), OFF and ON therapy.
