# Supplementary figures and images for: l-DOPA and Freezing of Gait in Parkinson’s Disease: Objective Assessment through a Wearable Wireless System
Source: Front Neurol. 2017 Aug 14;8:406. doi: 10.3389/fneur.2017.00406 (PMC5557738; doi:10.3389/fneur.2017.00406)

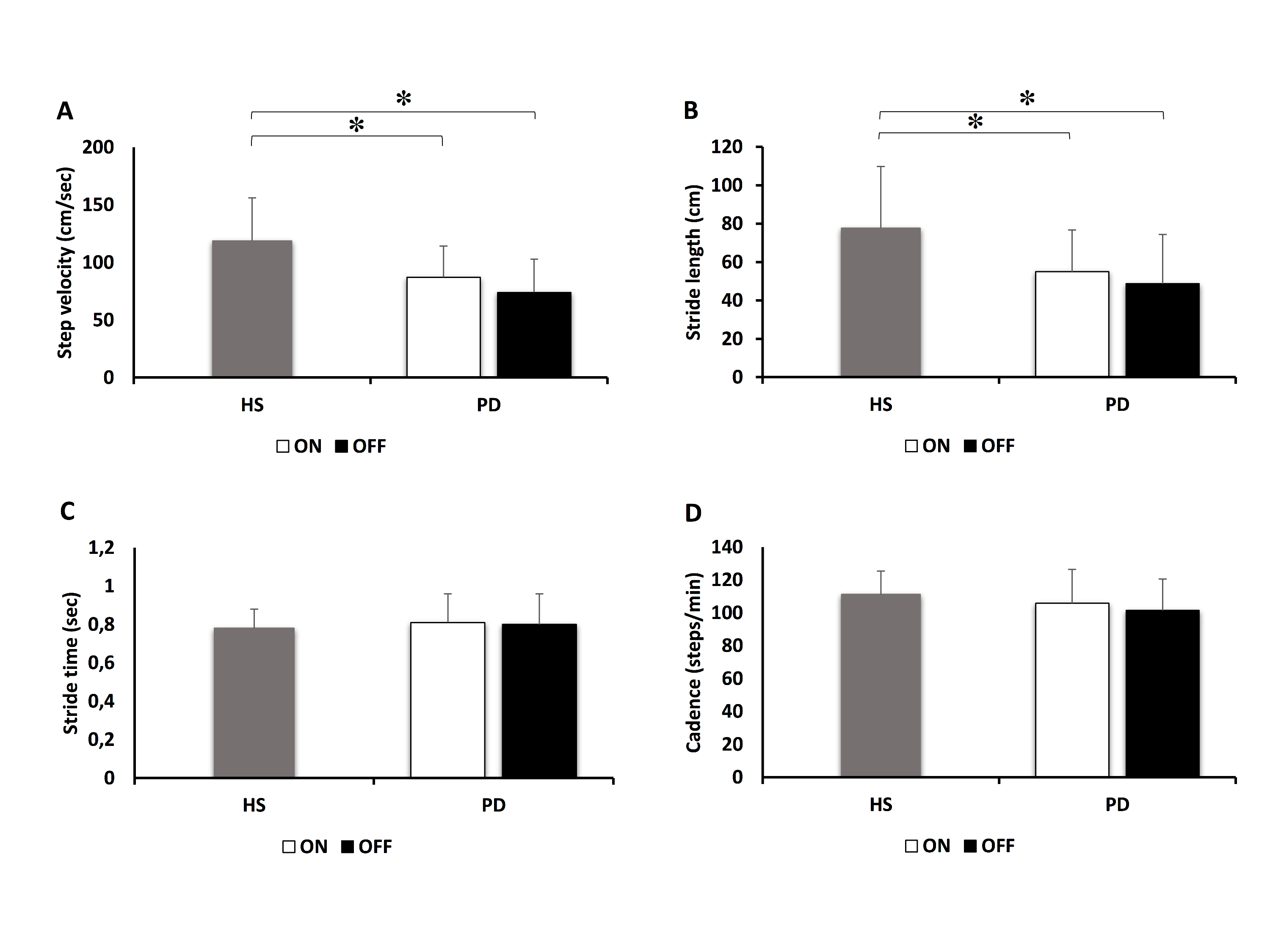

Supplement: Supplementary file 2 [file image_1.tiff]

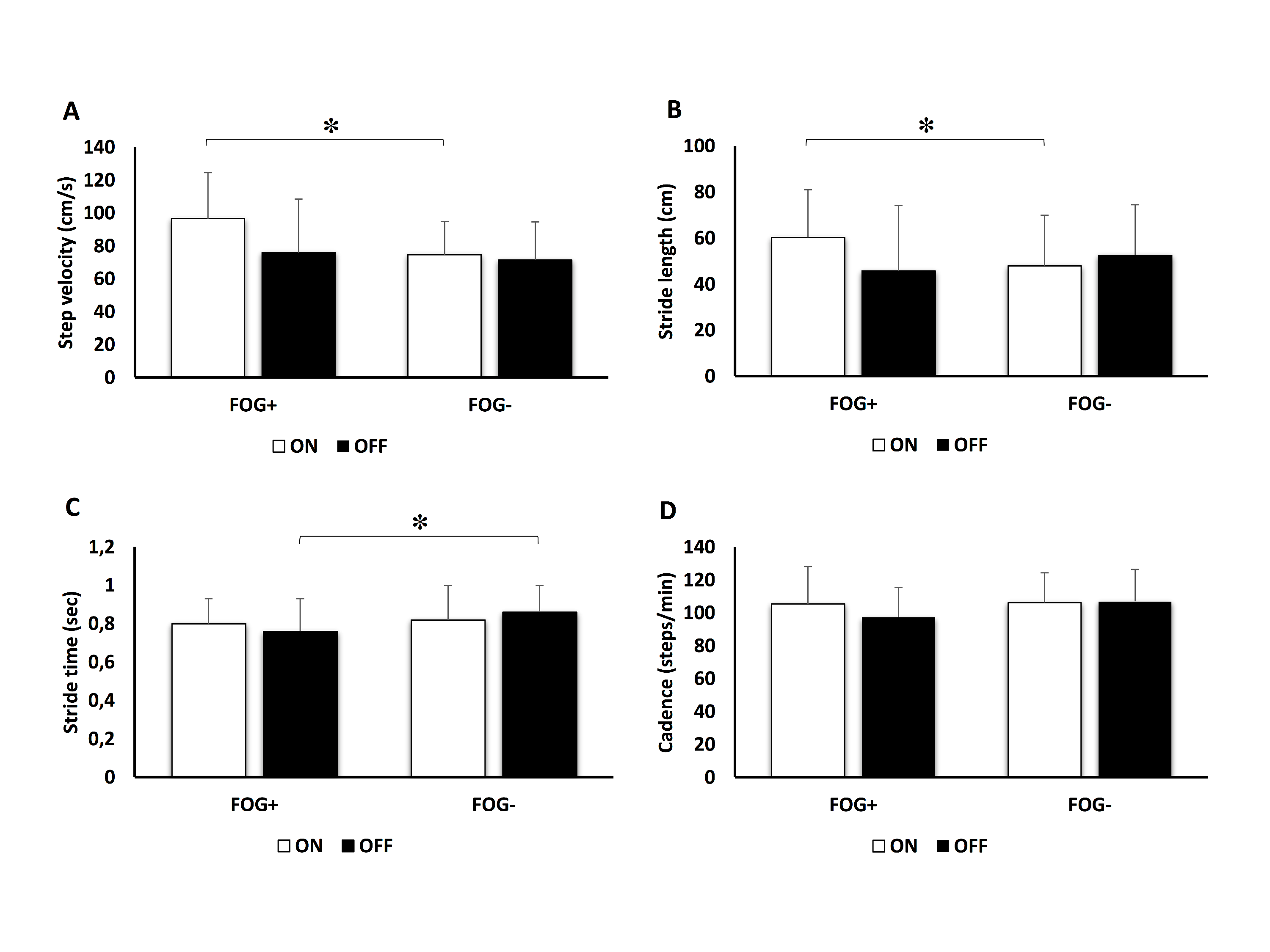

Supplement: Supplementary file 3 [file image_2.tiff]
